# An analysis of orphan medicine expenditure in Europe: is it sustainable?

**DOI:** 10.1186/s13023-019-1246-7

**Published:** 2019-12-11

**Authors:** Jorge Mestre-Ferrandiz, Christina Palaska, Tom Kelly, Adam Hutchings, Adam Parnaby

**Affiliations:** 1 Independent economics consultant, Madrid, Spain; 2 Dolon Ltd, London, UK; 3 Celgene International, Boudry, Switzerland

**Keywords:** Rare disease, Orphan medicinal product, Orphan medicines expenditure, Healthcare sustainability

## Abstract

**Background:**

Orphan medicinal product (OMP) prices are considered by some to be a challenge to the sustainability of healthcare expenditure. These concerns are compounded by the increasing number of OMPs receiving marketing authorisation (MA) annually. The aim of this study was to explore the sustainability of OMP expenditure within the context of total European pharmaceutical expenditure.

**Methods:**

Using historical IQVIA data, an analysis was conducted on total pharmaceutical and OMP expenditure in eight countries (using values / volumes) in the branded, non-branded and overall pharmaceutical market. Country level and aggregated data was considered for EU5 countries, Austria, Belgium and Ireland.

Three key analyses were conducted:

1. The OMP share of total pharmaceutical expenditure was calculated from 2000 to 2017, to assess its evolution over time.
2. The results of this analysis were compared with a 2011 forecast of OMP budget impact.
3. The evolution of the total pharmaceutical market and its different segments (branded OMPs, non-OMP branded and unbranded) were assessed by estimating the compound annual growth rate (CAGR) and percentage of pharmaceutical expenditure for each market segment from 2010 to 2017.

**Results:**

Across countries, OMP share of total pharmaceutical expenditure has increased each year since 2000, rising to 7.2% of total pharmaceutical expenditure in 2017. OMP expenditure has increased at a CAGR of 16% since 2010. The number of OMPs receiving MA each year showed a CAGR of 11% since 2001, four percentage points greater than the CAGR for all medicines receiving MA over the same period. OMP share of total pharmaceutical expenditure is higher than forecasted in 2011 due to slower than expected growth in the non-OMP market. OMP growth has been offset by reduced expenditure in the general market and increased use of generics and biosimilars.

**Conclusions:**

Relative spending on OMPs has increased over the last 20 years, but this has been largely compensated for within the current allocation of total pharmaceutical spending by flat expenditure for non-OMPs and increased volumes of (lower-priced) generics/biosimilars, reflecting a shift towards expenditure in higher cost, lower volume patient populations and a shift in drug development towards more specialised targeting of diseases.

## Background

Medicine prices are under increasing scrutiny by policymakers and are considered by some as a challenge to the financial sustainability of European healthcare systems [1–3]. In June 2016, the European Council concluded that: *“… new medicinal products however may also pose new challenges to individual patients and public health systems, in particular regarding the assessment of their added value, the consequences for pricing and reimbursement, [and] the financial sustainability of health systems …*” [4].

These concerns are also expressed specifically in the context of orphan medicinal products (OMPs), medicines approved for the treatment of rare diseases [3, 5–8]. Between 2011 and 2016, the number of OMPs obtaining European marketing authorisation (MA) increased by 18% per year [7]. This increase in approvals, combined with high per patient prices, has led to worries amongst policy makers about the affordability of aggregate OMP expenditure [3, 5, 6]. More recently, the introduction of high-priced gene and cell therapies – many of which are also OMPs – has further focused attention on OMP expenditure [9–11].

Such concerns prompted the European Council to advise the European Commission (EC) to evaluate its OMP regulation (European Union (EU) Orphan Regulation (No 141/2000)) [12]. An evaluation of this legislation should be supported by robust analyses of the underlying forces and expenditure trends that the regulation has stimulated. While some studies have been conducted on OMP expenditures, for example in the EU5 (France, Germany, Italy, Spain, United Kingdom) related to costs per patient and indication and OMP costs compared to overall medicines spending [13], and with a country focus on aspects such as budget impact in the Netherlands [14] and impact of OMP pricing mechanisms in Belgium [15], there are, however, relatively few analyses describing European OMP expenditure since the introduction of the OMP regulation, in this fast and dynamic field [16].

One such analysis, however, can be found in the frequently cited 2011 paper by Schey et al. [17], who reviewed OMP sales data from 2000 to 2010 and sought to estimate the future budget impact of OMPs for 2011–2020. The forecast predicted that European OMP budget impact would peak in 2016 at 4.6% of total pharmaceutical expenditure. After 2016, budget impact was expected to plateau between 4 and 5% [17].

To the best of our knowledge, there have been few if any more current attempts to analyse OMP expenditure trends in the EU or their impact on the overall medicines budget. This paper therefore seeks to describe the actual observed expenditure on OMPs in Europe between the introduction of the regulation in 2000 and 2018 (focusing on its share of total pharmaceutical expenditure), compare this to the previous forecast of OMP expenditure and explore the factors that may be driving any variance with the previous forecast.

This analysis further sought to examine total OMP expenditure in the context of trends in total European pharmaceutical expenditure, including all branded and non-branded medicines.

Ultimately, the main focus of this analysis is on sustainability of OMP expenditure within the current distribution of total pharmaceutical expenditure*.* We acknowledge that there are relevant additional questions on the sustainability of total pharmaceutical expenditure, such as whether the distribution of costs and savings due to entry of generics/biosimilars should be different, and where savings could or should be re-allocated. While these questions are relevant and valid, they require separate extensive research and provision of evidence in and of themselves, and are thus beyond the scope of this paper.

A recent paper by Espin et al. (2017) [18] projected, after adjusting for list-to-net price differences, an annual growth rate of 1.5% for total pharmaceutical expenditure in Europe (until 2021), a rate Espin and colleagues [18] deemed sustainable, and lower than the projections at list prices. The question still remains as to whether policy makers would consider this a sustainable rate as well. In light of this, this paper seeks to examine underlying trends in OMP value, volume and share of total expenditure, in order to investigate whether this poses a challenge to healthcare systems on the basis of their current expenditure patterns. The analysis presented here is based on list prices, which implies the data will overestimate the value of the market for both orphan and non-orphan medicines. We raise this as a limitation of the study below.

## Methods

Historical sales data was acquired from the IQVIA MIDAS database for OMPs and total branded and unbranded pharmaceutical expenditure for Austria, Belgium, France, Germany, Ireland, Italy, Spain and the UK from 2000 to 2018. The MIDAS database covers retail and hospital products and records sales based on list prices, i.e. without discounts applied. Sales data was provided in two forms: value (euro) and volume (standard units). The volume of product sales can be seen as a proxy for volumes of patients treated and was therefore included within the scope of the analysis to help explain underlying trends in the broader market. The OMP dataset was provided at a product level on a quarterly basis, whereas the aggregated pharmaceutical market data was grouped by IQVIA’s innovation classification system and provided on an annual basis. The data was provided on September 25th, 2018, and included current OMPs on the European Medicines Agency (EMA) register, as well as products that had previously lost their orphan designation. The analyses were conducted on aggregated data across the eight European countries mentioned above.

For the purposes of this study, “OMP sales” also include sales of products that were once OMPs, but which have since lost orphan designation. Their inclusion is based on the assumption that their sales following designation withdrawal are a result of the position of strength that has been consolidated within the market prior to a product losing its orphan status. Therefore, the rationale behind the chosen approach was to align with the socioeconomic perspective of OMPs and their impact on the pharmaceutical market. Nevertheless, the impact of excluding sales of OMPs following the loss of their orphan designation is also provided when relevant (noting here the impact can be considerable).

The OMP data acquired was at a product level, therefore including sales of all licensed indications for a product, including both orphan and non-orphan indications. However, IQVIA does not provide a split of sales per indication (whether orphan-designated or not), so it was decided that all sales for multi-indication medicines (with some indications not being “orphan”) would be included within the study as “OMP sales”. As such, the sales of OMPs in the analysis are likely to be overestimated – although we comment in the discussion section on other issues that might affect whether “OMP sales” over, or under estimate the true OMP expenditure.

Three key analyses were conducted:

1. The OMP share of total pharmaceutical expenditure was calculated from 2000 to 2017, to assess how it has evolved over time.
2. The results of this analysis were compared with the previously forecasted budget impact by Schey et al. [17] from 2011, which covered up to 2020.
3. The evolution (in value and volume) of the total pharmaceutical market, and the different segments within it (branded OMPs, non-OMP branded and unbranded) was assessed.

Each analysis is discussed in turn.

### Understanding the evolution of the OMP share of total pharmaceutical expenditure

The annual observed OMP expenditure was identified and calculated as a percentage of annual total pharmaceutical expenditure. For each of the eight countries, the total sales for OMPs and total pharmaceutical expenditure were determined and summed to form an aggregated figure. The OMP share of total pharmaceutical expenditure was calculated from 2000 to 2017, to assess how it has evolved over time.

### Comparing the OMP share of total pharmaceutical expenditure with previous forecast

The results of the first analysis in this study, from 2010 to 2017, were compared with the previously forecasted budget impact, published by Schey et al. in 2011 [17]. The forecast estimated that total pharmaceutical expenditure would continue to grow at the rate identified by IMS (now IQVIA) in a ‘Market Prognosis’ report over the previous five years of 6.6% annual growth rate from 2010 [17]. The total market expenditure growth rate of the acquired dataset was calculated to assess how this compared with the previous estimate.

To investigate the impact of total market expenditure growth on the relative share of the OMP market, the forecast overall market growth rate of 6.6% was applied to the total market from 2010 and the OMP share as a percentage of total expenditure was re-calculated.

### Investigating dynamics of the pharmaceutical market

Sales in value and volume for the eight countries were classified into three distinct market segments for analysis alongside the total market:

- **OMP**: All branded OMP sales
- **Non-OMP branded**: Sales from products classified by IQVIA as ‘innovative branded’
- **Unbranded**: Sales from products classified by IQVIA under the following categories
  - Non-original branded
  - All unbranded (although noting we do not have specific data for individual generic/ biosimilar versions of OMPs)
  - Other
  - Unassigned

The compound annual growth rate (CAGR) and share of total pharmaceutical expenditure for each segment and the total, in value and volume, over the period 2010–2017 was determined.

To derive an analysis of the evolution of the market directly comparable to the previous forecast, the previously identified 6.6% annual growth rate was applied to the non-OMP branded and unbranded segments from 2010. The approach assessed how these areas of pharmaceutical expenditure compared with an estimate of what was forecasted. This assumes the market segments would have been predicted to follow the same level of growth as the total market. An estimate for the annual OMP market value was produced by applying the same 6.6% annual growth to total market expenditure from 2010. The value in euros that corresponded with the forecasted budget impact percentage for that year was then calculated.

## Results

Results for each of the three analyses undertaken are presented in turn.

### Evolution of the OMP share of total pharmaceutical expenditure

**Fig. 1:**
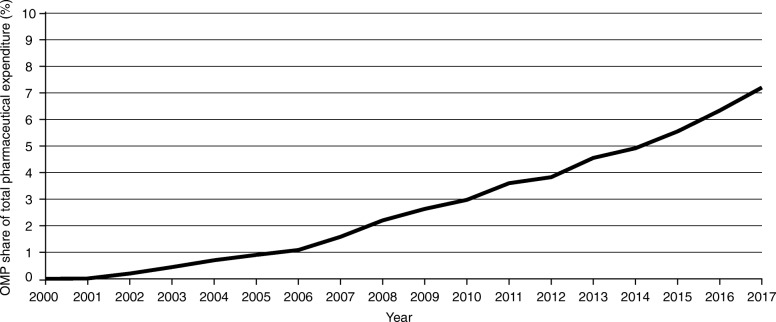
OMP share of total pharmaceutical expenditure for the eight countries aggregated (2000–2017)

As expected, given the increasing OMP authorisations, the OMP share of total pharmaceutical expenditure has increased consistently each year since 2000. Figure 1 displays the evolution of the aggregated OMP share of total pharmaceutical expenditure for the eight countries included. The OMP share was 7.2% in 2017, a result of OMP expenditure reaching approximately 10.5 billion euros against the approximately 147 billion euros spent in totality in medicines that year. Similar trends were observed across all countries (see Figs. 6, 7, 8, 9, 10, 11, 12 and 13 in the Appendix).

For the eight countries in total, OMP expenditure experienced a compound annual growth of 16% (2010–2017), ranging between 13 and 25% across individual countries (see Table 1), whereas total pharmaceutical expenditure experienced a compound annual growth of only 3% over the same time period. The number of OMPs receiving MA each year (from the EMA) has experienced a CAGR of 11% since 2001, but with high variance among individual years between 3 and 19 authorisations per year, for a total of around 150 (see Fig. 14 in the Appendix). In comparison, the CAGR of the total number of medicines receiving approval has been 7% since 2001. The proportion of MAs each year that is attributed to OMPs has risen since 2001, from 9 to 17% in 2017.

**Table 1:** EU8 market-specific OMP expenditure CAGRs from 2010 to 2017

	OMP EXPENDITURE CAGR (%)
AT	24.5
BE	14.1
DE	15.9
ES	13.2
FR	14.1
IE	15.6
IT	16.4
UK	21.8

### Comparing the OMP share of total pharmaceutical expenditure with the previous forecast

**Fig. 2:**
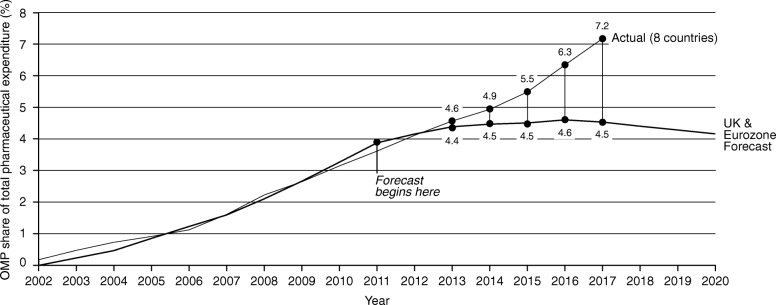
OMP share of total (actual) pharmaceutical expenditure vs. budget impact forecast

As can be seen in Fig. 2, between 2002 to 2013 the actual OMP share of total pharmaceutical expenditure was in line with the previous analysis’ observed values until 2010 and for the first few forecasted years until 2013. Following 2013, however, the OMP share of total pharmaceutical expenditure has been higher in reality than what was forecasted.

The forecast assumed a total market growth rate of 6.6%, which led to a predicted peak in orphan budget impact of 4.6% in 2016. In reality, the average total market annual growth rate has been lower (3.0%), which has contributed to the higher OMP market share of total expenditure (7.2%). In 2017, the OMP share of total pharmaceutical expenditure was 5.6% of adjusted total expenditure, versus 4.5% in the forecast.

If sales are only included for products which maintain their OMP status, the budget impact is reduced to lower than what was forecasted, peaking in 2017 at 4.5%. Excluding all sales following loss of orphan designation, however, likely underestimates OMP expenditure in this approach.

### Investigating dynamics of the pharmaceutical market

**Fig. 3:**
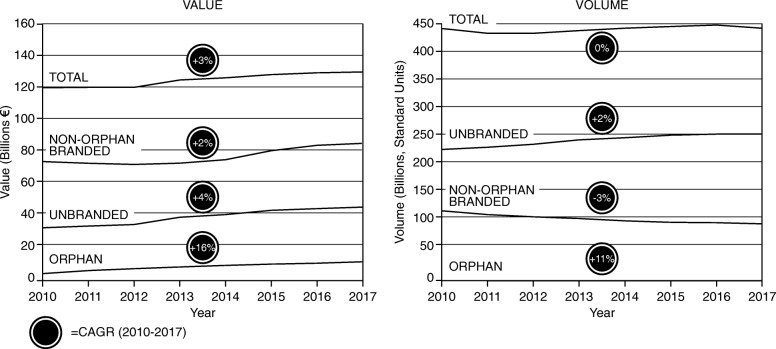
Aggregated market profile for the eight countries, displayed in value (left) and volume (right)

Despite the increase in OMP expenditure, this does not appear to be driving (additional) growth in the total market, as this is offset by changes in the broader market. Primarily, the balance between OMP, non-OMP branded and unbranded medicines remains the same, as can be seen in Fig. 3 (value and volume of branded vs. non-branded medicines), and Table 2.

**Table 2:** Market shares and their CAGRs, for the eight countries, from 2010 to 2017

	Market share (%)					
	Value			Volume		
	2010	2017	CAGR	2010	2017	CAGR
Orphan	3.1	7.2	13	< 0.1	< 0.1	11
Non-Orphan Branded	62.4	57.3	-1	27.6	22.6	-3
Unbranded	28.2	30.0	1	52.4	58.1	1
Other Expenditures^a^	6.3	5.5	-2	20.0	19.3	-1

^a^Other expenditures include over-the-counter (OTC) products and vaccines, which have been omitted from this analysis

Figure 3 displays the annual value and volume of OMP, non-OMP branded and unbranded medicines from 2010 to 2017 – noting the share for OMP in volume terms not showing explicitly due to low numbers. All of the market segments have experienced growth in value to varying degrees since 2010. There is, however, a growing divergence between the unbranded and non-orphan branded markets in sales volume, with the unbranded market growing and the non-orphan branded market sales declining over the years. Table 2 displays the share of total pharmaceutical expenditure and CAGRs of OMP, non-OMP branded and unbranded medicines from 2010 to 2017. The share of total pharmaceutical expenditure of OMP and unbranded have risen (but show a difference in magnitude), while that of non-OMP branded have declined in volume terms, and have grown the least in value terms.

**Fig. 4:**
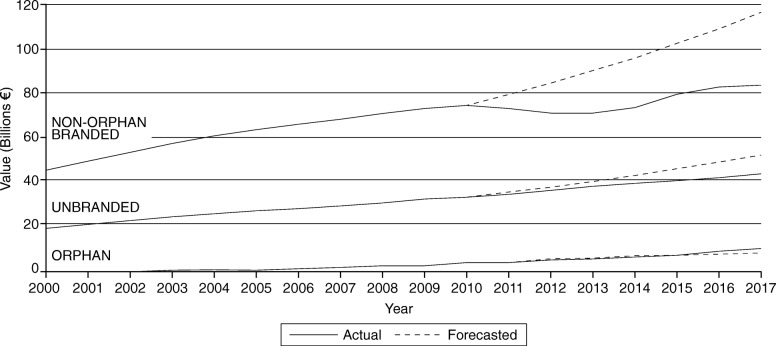
Actual market value vs. forecasted OMP budget impact and non-OMP branded/unbranded market growth

On the one hand, the growth of the non-OMP branded market has slowed considerably, resulting in a reduction in its market share (relative to the total market) both in value and volume terms. On the other hand, the unbranded segment’s share has grown, albeit at a small rate. From 2010 to 2017, OMP volume has grown by 0.02 percentage points, while non-OMP branded has declined by 5 percentage points, and unbranded has increased by 5.7 percentage points (See Table 2). Figure 4 below shows this overall picture by comparing the forecasted OMP, non-OMP branded and unbranded medicine market values to what is being observed in reality (difference between solid and dashed lines) – the biggest discrepancy is observed for the “non-OMP branded” segment.

## Discussion

This analysis aimed to understand the observed expenditure on OMPs in Europe between the introduction of the EC regulation in 2000 and 2018 by investigating the factors driving spending, and how expenditure on OMPs fits within total European pharmaceutical expenditure. In particular, the primary focus was understanding if OMP expenditure could be deemed as sustainable within the current distribution of total pharmaceutical expenditure.

The consistent annual growth in the OMP market since 2010 with a decreasing share of non-OMP branded medicines and increasing overall share of OMPs, may reflect an underlying change in the structure of branded expenditure. This trend suggests a shift in medicines expenditure to more complex diseases with smaller patient populations and higher unmet need. This shift appears to have been compensated by the savings being made from non-OMP branded medicines facing generic competition, as suggested by the increased volume of non-branded (i.e. generic as opposed to non-orphan branded) medicines in a total market with a stable volume.

Similar trends have been observed in the United States; spending has shifted towards specialty medicines that treat comparatively few people with chronic or rare diseases. Specialty medicine spending in the U.S. has increased from 11% in 1997 to 43% in 2017 [19]. Within the same time period, OMP spending (of which 87% falls under the label of speciality medicines) increased from 4 to 10% [19]. Another factor favouring the OMP market has been the (financial and non-financial) incentives provided by the EC, which was the purpose of the regulation in the first place.

The growth rate of the number of new OMPs authorised is smaller than that of OMP expenditure. This could be because the average price (or cost per patient) for OMPs is higher than for non-OMPs, or because the number of patients treated per OMP might have increased. Further research is required, however, to determine the exact cause for this result.

The findings of the second key analysis initially suggested a misalignment between what was forecasted in 2011 by Schey et al. and what was observed in this study. However, once the observed data was adjusted to compensate for the forecast’s overestimation of the total market growth rate, it was much more aligned with the forecast than it originally appeared to be. In the methodology of the Schey et al. analysis, different approaches were used to predict future orphan expenditure and future total market pharmaceutical expenditure. A bottom-up approach was taken to forecast orphan drug expenditure, based on predictions of the number of new orphan drugs. For the forecast of total market pharmaceutical expenditure, a linear extrapolation approach was used, based on the historical annual growth rate of 6.6% mentioned earlier in this paper. Given the findings of our analysis, it appears that the bottom-up approach was more accurate than the linear extrapolation approach, and could therefore be considered for future forecasts.

Furthermore, there are three differences in the methodology/data used in the two analyses: (i) the forecast looked only at the orphan indications of medicines, in this analysis both OMP and non-OMP medicine indications were included; (ii) in the forecast, savings from the entry of biosimilars were assumed, and this has not been observed in the market to date; (iii) in the last seven to 10 years there has been an increased use of discounts and rebates for OMPs, and the gap between the list and net price has increased for specialty medicines [18]. This gap, which was about 1.4 percentage points in the study by Espin et al. (2.9% list growth vs. 1.5% net growth) [18] is not captured in this analysis. It is true, however, that list prices are also used for non-OMP expenditure, but it seems that the level of (confidential) discounting and rebates is higher for medicines used in hospitals rather than dispensed in pharmacies, and OMPs are usually used in hospital settings [18, 20]. Therefore, observed total expenditure will be overestimated within this study, especially for more recent years. Within this context, the OMP share of total pharmaceutical expenditure is more aligned with the predicted budget impact. It is also important to note, however, that there is uncertainty in how the raw data used in both Schey et al. and in this paper truly reflect actual use of/expenditure on OMPs, and it is not possible without further analyses to quantify the impact of each the three differences just explained.

The third key finding highlighted some of the complex dynamics of the pharmaceutical market, and the factors that may enable health systems to increase expenditure on medicines for disease areas with higher unmet need, like OMPs. Many factors will play a role in the changing dynamics and in the significant growth rate of OMPs, but one of them may reflect the response of the pharmaceutical industry to the OMP regulation by investing in researching and developing OMPs. Indeed, it has been estimated that as of 2017, OMP-designated projects represented 13% of all products in clinical development, and that these percentages were even higher for the later stages of development (17 and 22% in Phase III and Regulatory Review respectively) [21]. An additional factor may be that policymakers do not necessarily scrutinise the large overall increase in OMP spending, due to the relatively small budget impact of individual products.

Another critical factor underpinning the dynamics of the market is the impact of patent expirations. For non-OMPs in particular, there has also been an increasing volume of use of biosimilars and generics, resulting in a decreasing use of branded medicines (as a proportion of total use) [22]. In many cases the biggest drivers for savings come as a result of the decrease in prices of off-patented medicines. The impact of patent expiry and higher use of generics is expected to be 37% larger between 2018 and 2022 than the previous 5 years [23].

For branded OMPs in particular, the potential impact of generics/biosimilars could decrease the economic burden. In the U.S., this impact has been documented: of 503 drugs with orphan designation, 217 have lost their patent protection, and 116 have generic competitors [24]. This raises the question as to whether there is any form of generic competition with OMPs in Europe (including biosimilars), and if so, what is the repartition of such competition among the therapeutic areas and what are the contributing factors (including price). This is beyond the remit of this study, but it has been argued that, compared to generics, biosimilars are generally harder to develop and manufacture (possibly even harder for OMP biosimilars), which is partly why there are generally less competitors, and thus less (expected) competitive pressure; another barrier to entry includes possible physician reluctance to use biosimilars.^1^ Moreover, the database used for this paper does not contain sales/use of generic/biosimilar versions of OMPs.

**Fig. 5:**
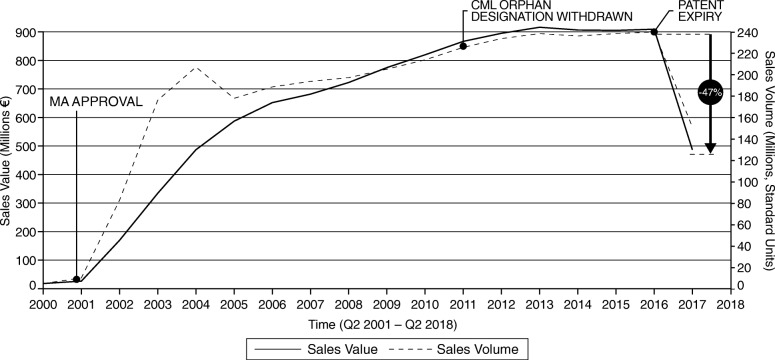
Case study example: Glivec expenditure 2000–2017

**Fig. 6:**
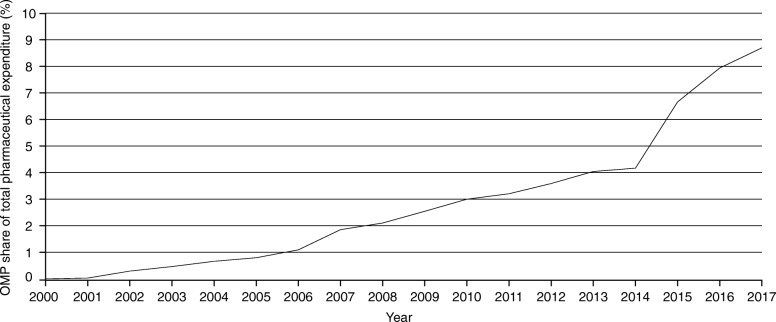
OMP share of total pharmaceutical expenditure in Austria (2000–2017)

**Fig. 7:**
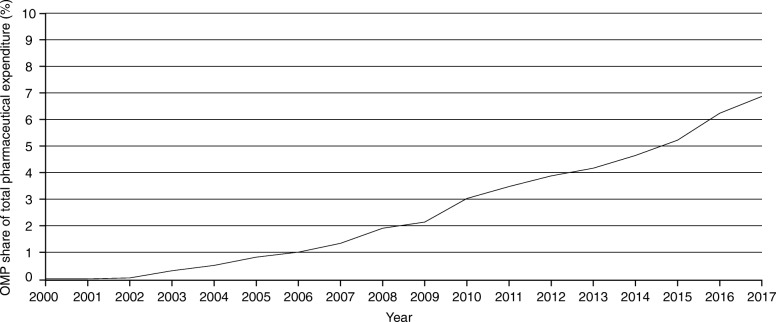
OMP share of total pharmaceutical expenditure in Belgium (2000–2017)

**Fig. 8:**
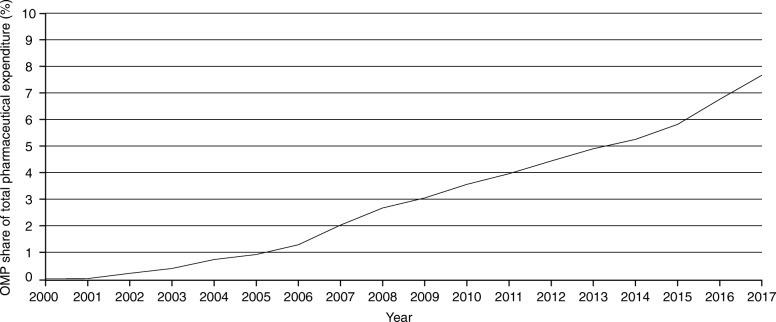
OMP share of total pharmaceutical expenditure in Germany (2000–2017)

**Fig. 9:**
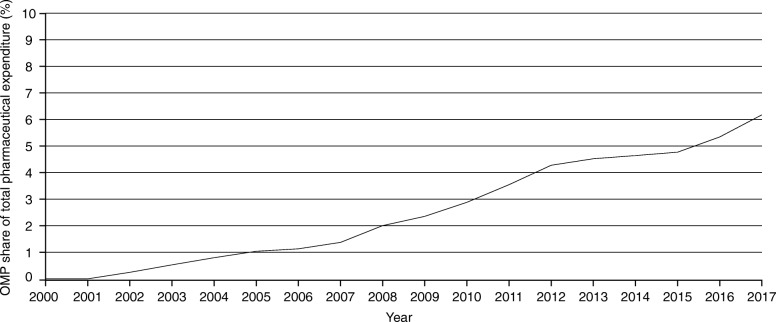
OMP share of total pharmaceutical expenditure in Spain (2000–2017)

**Fig. 10:**
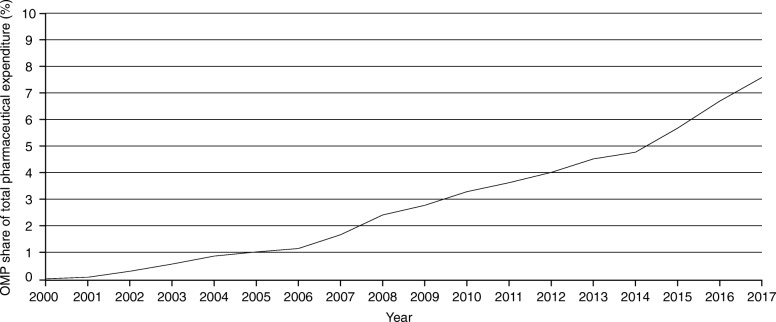
OMP share of total pharmaceutical expenditure in France (2000–2017)

**Fig. 11:**
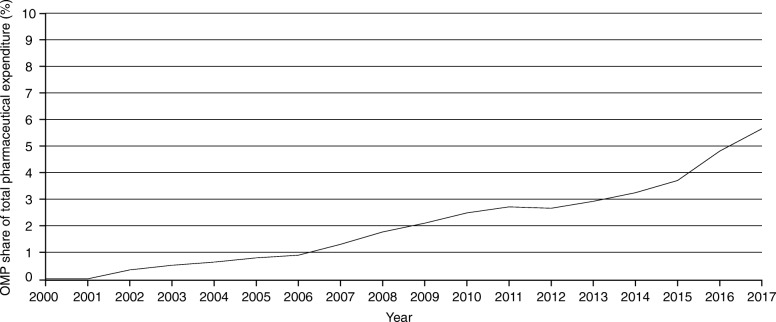
OMP share of total pharmaceutical expenditure in Ireland (2000–2017)

**Fig. 12:**
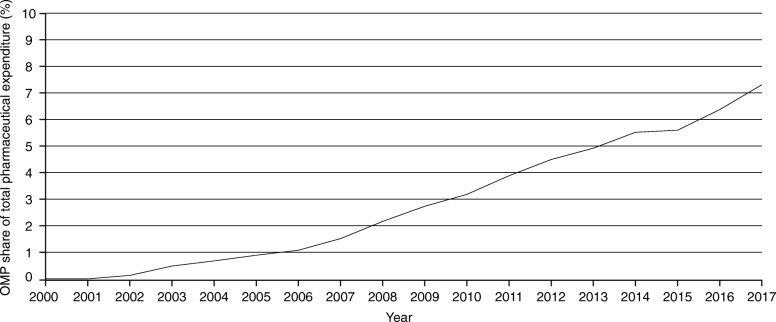
OMP share of total pharmaceutical expenditure in Italy (2000–2017)

**Fig. 13:**
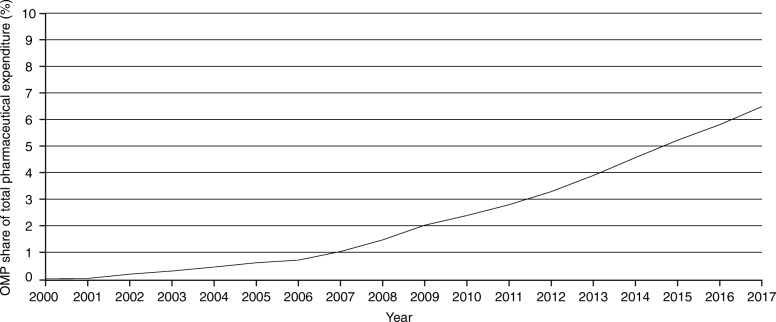
OMP share of total pharmaceutical expenditure in the United Kingdom (2000–2017)

**Fig. 14:**
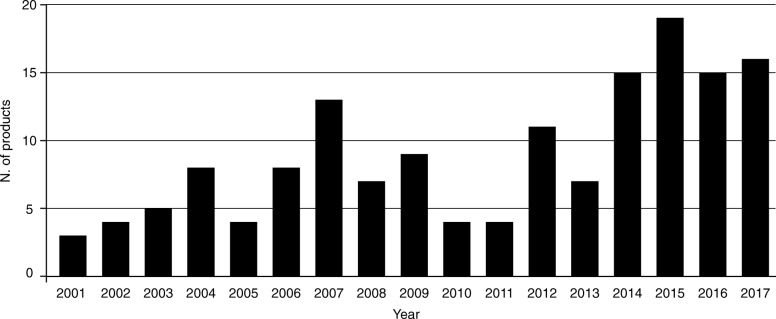
Number of study sample OMPs receiving MA per year (2000–2017)

As data for specific branded OMPs is, however, included in the database, the evolution of sales of one OMP example is presented (Fig. 5). It is important to emphasise that this is only one example (Glivec, the small molecular kinase inhibitor imatinib) and was not part of the main analyses of this study. It is included here as it displays one possible scenario to estimate the impact of generic/biosimilar versions of OMPs, bearing in mind that the product in question was the OMP with highest sales during the period 2002–2015 across all OMPs that had orphan designation during the same time period – and the empirical literature highlights, among other things, that size of the market is an important driver of generic/biosimilar entry and competition more generally. Figure 5 highlights, where applicable, critical milestones (MA approval, orphan designation withdrawal (2011) and patent expiry (2016)) for that specific product. Glivec also lost its marketing exclusivity in 2011 for its chronic myeloid leukemia indication. As displayed in Fig. 5, following the patent expiry of imatinib at the end of 2016, a decrease in annual expenditure of 47% had been observed by 2017, which can be considered significant.

More generally, the shift in volume from branded to generics has been a stabilising factor in the pharmaceutical market [22], and the question remains what could/should be the potential impact from generic/biosimilar competition for OMPs in Europe. The experience of Glivec shows an important effect, albeit it is unclear how representative this example will be for other OMPs in the near future, especially for OMPs with (significantly) lower sales. Nevertheless, the impact of generic/biosimilar entry needs to be examined across all OMPs, and further research on potential challenges for the entry of biosimilars for OMPs is also necessary to investigate this issue more thoroughly, building from previous analyses looking at drivers of generic/biosimilar competition (for more information, see, for example, the work commissioned by the EC on supplementary protection certificates, pharmaceutical incentives and rewards in Europe^2^).

Aggregate expenditure on OMPs appears not to be increasing total pharmaceutical expenditure, but questions remain regarding the efficiency and equity of increasing spending on patients with rare diseases. Drummond and Towse [25] outlined challenges with OMP funding that relate to the numerous necessary points of consideration, such as during pricing and reimbursement negotiations/processes, which they suggest need to be addressed with policy improvements. Such points of consideration include, for example, the values and objectives of society; ensuring that cost and profit of OMPs are reasonably equivalent to that of other drugs to avoid them being disproportionately costly or profitable; a clear definition of research priorities for OMPs and strong clarification of the OMP designation to ensure incentives are not exploited, i.e., to ensure investment is directed toward the resources to develop treatments for diseases deemed of highest priority; and international collaboration among governments to increase the small number of patients per country [25].

The relative value of expenditure on OMPs versus non-orphan branded is critical to understanding the efficiency of the changes in overall expenditure. Perceptions of the value of OMPs may be influenced by the levels and types of evidence of clinical benefit. Evidence development in rare diseases poses specific challenges; small, heterogeneous populations and diseases of which little natural history is known, make it difficult to produce clinical evidence of the standard required for other medicines [26]. At the same time, there is a ‘polyphony that exists … about the acceptability or not of individual pieces of evidence’ [27]. That is, some health technology assessment (HTA) agencies are willing to accept lower quality evidence of OMPs, or have higher willingness to pay thresholds, while others do not allow such lenience in evidence generation for OMPs. This variation in standards of acceptability of evidence leads to some HTA agencies accepting the value of OMPs approved on the basis of surrogate endpoints, whilst some commentators dismiss their value without further analysis [5]. This is underscored by the high cost of OMPs (per patient), and the need for consistent evaluation processes that are practical and sustainable. Efforts to improve HTA infrastructure that accounts for the ‘feasibility and acceptability’ of evidence and explicitly addresses the uncertainty associated with products with less mature or earlier phase data has potential to address these issues.

Four limitations of this study should be acknowledged, which have an impact on whether the estimated share of total pharmaceutical expenditures fully reflects OMP use in practice. First, IQVIA (source of use and sales data) might not fully capture the use of OMPs in countries, as sometimes such products are accessed via other routes, such as compassionate use. Second, while the focus on the eight countries produces representative results of western European countries, there are of course other European countries with their own processes for OMPs, which were not included in the analysis. The use of OMPs in these other countries may be higher or lower than these eight countries. Third, and as mentioned before, non-OMP indications were included in the analyses (as “OMP sales”), due to not being able to separate out the data (either because some OMPs were no longer OMP-designated, or because it included sales for non-OMP indications for a medicine with at least one OMP-designated indication). This implies the estimates of OMP sales presented here would be an overestimate. Fourth, the analyses are based on expenditure at list prices, which overestimates the value of the market. However, to our knowledge there is no information available comparing the level of discounting between OMPs and non-OMPs, although confidential discounts have been shown to be greater for medicines used in a hospital setting [18, 20].

Despite these limitations, the findings from these analyses provide insight into current OMP expenditure in the context of broader expenditure trends in the branded and unbranded pharmaceutical market. Both these segments are moving in opposite directions, but ultimately “balancing out” to provide a relatively stable growth rate overall. This study suggests that in recent years the increasing expenditure on OMPs has been offset by slower growth in the wider market. Although it could therefore be argued that the market has been financially sustainable, uncertainty exists as to whether the observed trends will continue to be sustainable not only financially, but also politically.

## Conclusion

The EU regulation on OMPs has had success in fostering R&D addressing unmet needs for treatments for rare diseases, with approximately 150 OMPs being approved since its introduction. However, despite the success of the regulation in supporting the development of medicines for rare diseases, the findings from this analysis suggest that the resultant impact on OMP expenditure could be deemed sustainable when seen in the context of total pharmaceutical expenditure. The drivers of expenditure shares across the different components of the total market, however, might be changing, which implies different future dynamics relative to past dynamics. There are signs that the market is undergoing a shift towards higher cost, lower volume medicines in patient populations with high unmet need, and more specialised targeting of diseases, all of which is compensated by increased volumes of (cheaper) generics and flat expenditure for non-OMP (branded) medicines for diseases in which more treatments exist. Future research should seek to further investigate the impact that the entry of biosimilar/generic products will have on the OMP market and future savings. In addition to in-depth analysis of expenditure trends, it is important to look critically into the detailed processes of incentives, and pricing and reimbursement of OMPs, to work towards ensuring long term stability, while at the same time providing the “right” incentives to (keep) encourage(ing) R&D in this area. Any changes to legislation require a strong understanding of underlying trends. This analysis sought to contribute to that understanding, and findings suggest that when the situation as a whole in terms of market growth and use of biosimilars/generics is taken into account, concerns about OMP market expenditure might be alleviated.

## Data Availability

The data that support the findings of this study were provided by IQVIA, but restrictions apply to the availability of these data, which were used under license for the current study, and so are not publicly available.

## Abbreviations

AT: Austria
BE: Belgium
CAGR: Compound annual growth rate
DE: Germany
EC: European Commission
EMA: European Medicines Agency
ES: Spain
EU: European Union
FR: France
HTA: Health technology assessment
IE: Ireland
IT: Italy
MA: Marketing authorisation
OMP: Orphan medicinal product
OTC: Over-the-counter
UK: United Kingdom

## Footnotes

1 For the interested reader, see for example, Mestre-Ferrandiz, J., Towse, A. and Berdud, M. 2016, Biosimilars: achieving long-term savings and competitive markets. GaBI Journal, 5(3), pp. 1–3

2 https://ec.europa.eu/docsroom/documents/29521
